# Deep learning for named entity recognition on Chinese electronic medical records: Combining deep transfer learning with multitask bi-directional LSTM RNN

**DOI:** 10.1371/journal.pone.0216046

**Published:** 2019-05-02

**Authors:** Xishuang Dong, Shanta Chowdhury, Lijun Qian, Xiangfang Li, Yi Guan, Jinfeng Yang, Qiubin Yu

**Affiliations:** 1 Center of Excellence in Research and Education for Big Military Data Intelligence (CREDIT), Department of Electrical and Computer Engineering, Prairie View A&M University, Texas A&M University System, Prairie View, Texas 77446, United States of America; 2 Schools of Computer Science and Technology, Harbin Institute of Technology, Harbin, China; 3 Schools of Software, Harbin University of Science and Technology, Harbin, China; 4 Second Affiliated Hospital of Harbin Medical University, Harbin, China; USC Information Sciences Institute, UNITED STATES

## Abstract

Specific entity terms such as disease, test, symptom, and genes in Electronic Medical Record (EMR) can be extracted by Named Entity Recognition (NER). However, limited resources of labeled EMR pose a great challenge for mining medical entity terms. In this study, a novel multitask bi-directional RNN model combined with deep transfer learning is proposed as a potential solution of transferring knowledge and data augmentation to enhance NER performance with limited data. The proposed model has been evaluated using micro average F-score, macro average F-score and accuracy. It is observed that the proposed model outperforms the baseline model in the case of discharge datasets. For instance, for the case of discharge summary, the micro average F-score is improved by 2.55% and the overall accuracy is improved by 7.53%. For the case of progress notes, the micro average F-score and the overall accuracy are improved by 1.63% and 5.63%, respectively.

## Introduction

Electronic Medical Record (EMR) [1], a digital version of storing patients’ medical history in textual format, has shaped our medical domain in such a promising way that we can gather all information into one place for healthcare providers. To construct a comprehensive system to process EMR, we need different modules such as word-level modules including Part-of-Speech (POS) and Named Entity Recognition (NER), sentence-level modules like dependency parsing and semantic role labeling, and document-level modules, for example, classification and summarization. Typically, these different modules need different models. For the EMR summarization, the EMR is summarized from two dimensions: extractive summaries and abstractive summaries [2]. Modules such as CliniViewer [3] and IHC Patient Worksheet [4] were built. For the document classification, extracted information from EMR is used to predict heart failure [5] and suicide risk stratification [6] by building deep learning models [7] such as DeepPatient [8], Doctor AI [5], and eNRBM [6]. Specifically, unstructured data in EMR presents patients’ health condition and information such as symptoms, medication, and disease, where the information facilitates medical specialists and providers to track digital information and monitor them for patients’ regular check-up. Therefore, information extraction [9] from EMR is one of the most important tasks in medical domain. However, to extract information like medical named entities is labor intensive and time consuming. Moreover, adopting current models for the purpose of medical entity recognition from EMR has been demonstrated as a challenging task, because most of the EMRs are hastily written and incompatible to preprocess [9]. In addition, incomplete syntax, numerous abbreviation, units after numerical values make the recognition task even more complicated [10]. Standard Natural Language Processing (NLP) tools cannot perform efficiently when they are applied on EMR, since the entity terms of standard NLP is not designed for medical domain. Therefore, it is necessary to develop effective method to perform entity recognition from EMR.

In recent years, various deep learning based methods have been developed for Named Entity Recognition (NER) [11] from EMR. Recurrent Neural Network (RNN) such as Long Short-Term Memory (LSTM) is taking prominent place in NER due to its ability of dependency building in neighboring words. Wang et al. [12] studied bi-directional LSTM architecture and concluded that this model is very effective for predicting sequential data. Moreover, the performance of the model is not based on language dependency. Simon et al. [13] and Vinayak et al. [14] used bi-directional RNN model on their Swedish EMR and Hindi dataset, respectively. Similarly, the approach of using bi-directional RNN with LSTM cell has proven to perform well in named entity recognition task [15]. Futhermore, Lample at al. [16] combined CRF with bidirectional LSTM RNN to build LSTM-CRF for accomplishing NER, where words were represented as word embeddings to feed the bidirectional LSTM RNN, and new features generated by bidirectional LSTM RNN were as input to CRF to complete NER. Compared to LSTM-CRF, Ma et al. [17] introduced convolutional neural networks (CNN) to enhance the wordembeddings by extracting character-level representations of words. Peng et al. [18] built a joint model by implementing a multitask learning method to learn word segmentation and NER simultaneously based on LSTM-CRF. Yang et al. [19] explored the problem of transfer learning for neural sequence taggers to relieve the lacking of annotated data in some domain, where a source task with plentiful annotations (e.g., POS tagging on Penn Treebank) is used to improve performance on a target task with fewer available annotations (e.g., POS tagging for microblogs). For NER on Chinese EMR, Dong et al. [20] present deep transfer learning model with LSTM RNN for NER on Chinese EMR. Chowdhury et al. [21] propose a multitask bidirectional LSTM RNN to enhance mining medical terms from EMR. In both cases, the model demonstrated better performance comparing to the state-of-the-art model. Additionally, Convolutional Neural Network (CNN) model is used for improving NER in EMR [22–24]. Furthermore, a hybrid LSTM-CNN is proposed in [25], where the CNN is used to extract the features and fed them to LSTM model for recognizing entity types from CoNLL2003 dataset.

**Fig 1:**
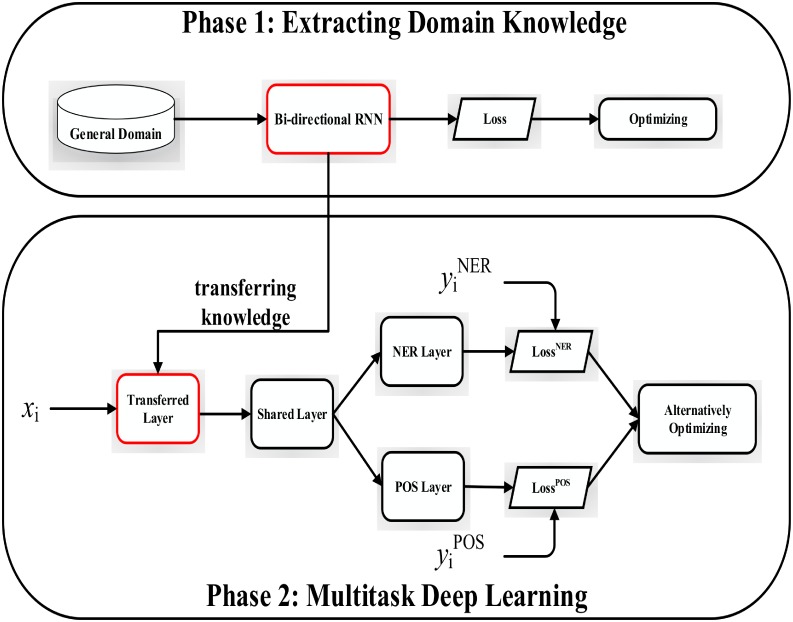
Framework of the proposed model for NER.

In general, training deep learning models requires large corpus datasets in order to estimate huge mount of model parameters accurately. However, there are limited number of available corpus of EMR that hinders the development of NER. Moreover, building labeled Chinese EMR data faces many challenges [26], and most organizations will not share their data publicly as the data contains private information of patients. In order to address these challenges, we combined deep transfer bi-directional RNN with multitask bi-directional RNN model to extract medical terms from Chinese EMR, since both deep transfer learning [20] and multitask deep learning show their potentials to strengthen NER performance. Building the proposed model needs two steps. In the first step, we obtain the general knowledge for NER in the general domain by training a bidirectional RNN on Chinese corpus. The second step is to transfer the general knowledge to construct a multitask bidirectional RNN on the Chinese EMR corpus. It is motivated by the observation that the performance of multitask learning model and deep transfer learning is much better comparing to individual learning approach when there is limited corpus dataset [20, 27]. The framework of the proposed multitask transfer bi-directional RNN model for NER is given in Fig 1.

In summary, the contributions of this study are as follows:

- A novel scheme of combining deep transfer learning and deep multitask learning is proposed for enhancing NER on Chinese EMR by using bidirectional LSTM RNN [16–18] and transfer learning technique [19, 20]. To the best of our knowledge, it is the first attempt to combine these two methods to improve the performance of NER on Chinese EMR. The proposed scheme has great potentials to improve performance of other NLP tasks such as dependency parsing and text classification.
- We validate our proposed scheme by testing on the discharge summary and progress note datasets, and evaluate the experimental results with different evaluation metrics. The evaluation results demonstrate the proposed scheme could enhance NER accuracy on the discharge summary datasets significantly.

## Materials and methods

The EMR dataset used in our experiment was collected from the departments of the Second Affiliated Hospital of Harbin Medical University, and the personal information of the patients have been discarded. An annotated/labeled corpus consisting of 500 discharge summaries and 492 progress notes has been manually created. The EMR data are written in Chinese with 55,485 sentences. The annotation was made by two Chinese physicians (A1 and A2) independently [24, 26]. It is categorized into five entity types: disease, symptom, treatment, test, and disease group.

In this work, a novel bi-directional RNN model is proposed for extracting entity terms from Chinese EMR. The proposed model can be divided into two phases: extracting domain knowledge and multitask learning phase, see Fig 1. In the first phase, we train a bidirectional LSTM RNN in the general domain. We select the optimal hyper-parameters such as learning rate and batch size to obtain highest accuracies on mining named entities from the general domain. Then, we assume that the knowledge could boost the performance of NER in a specific domain and transfer the knowledge to complete the NER on Chinese EMR, where the knowledge presents in the bidirectional layers learned in the first phase. In the second phase, we transfer the knowledge to the multitask deep learning by initializing the transferred layer as the appropriate knowledge could be employed to improve accuracies of NER on Chinese EMR [20]. Next step is to multitask bidirectional LSTM RNN. In this step, we fine tune the transferred layer on the Chinese corpus of EMR. The output of the transferred layer is input to the shared layer in order to extract more accurate relations between words. Then these relations are shared by two different task layers, namely the parts-of-speech tagging task layer and the named entity recognition task layer. These two tasks layers are trained alternatively so that the knowledge learned from named entity recognition task can be enhanced by the knowledge gained from parts-of-speech tagging task. Specifically, vector representation of each word in both of phases is a concatenation of word embedding and character embedding.

RNN [28] is an artificial neural network which can capture accurate item relations in sequences such as sentences. It could compute each word of input sequence (*x*_1_, *x*_2_, ⋯, *x*_*n*_) and transforms the sentence into a vector form (*y*_*t*_) by using the following equations:

$$\begin{array}{c} h_{t}=H\left(Ux_{t}+Wh_{t-1}+b_{h}\right). \end{array}$$ (1)

$$\begin{array}{c} y_{t}=Vh_{t}+b_{y}. \end{array}$$ (2)

where *U*, *W*, *V* denote the weight matrices of input-hidden, hidden-hidden and hidden-output processes, respectively. *h*_*t*_ is the vector of hidden states that derive the information from current input *x*_*t*_ and the previous hidden state *h*_*t*−1_.

Compared to RNN, the bi-directional RNN [29] is able to exploit both past and future context, where forward hidden states compute forward hidden sequence while backward hidden states compute backward hidden sequence. The output *y*_*t*_ is generated by integrating the two hidden states. The whole procedure is given by the following equations.

$$\begin{array}{c} h_{t}^{1}=H\left(U^{1}x_{t}+W^{1}h_{t-1}+b_{h}^{1}\right). \end{array}$$ (3)

$$\begin{array}{c} h_{t}^{2}=H\left(U^{2}x_{t}+W^{2}h_{t-1}+b_{h}^{2}\right). \end{array}$$ (4)

$$\begin{array}{c} h_{t}=h_{t}^{1}+h_{t}^{2}. \end{array}$$ (5)

$$\begin{array}{c} y_{t}=Vh_{t}+b_{y}. \end{array}$$ (6)

where *U*^1^, *W*^1^, *V*^1^ denote the weight matrices of the positive time direction while *U*^2^, *W*^2^, *V*^2^ denote the weight matrices of the positive time direction, respectively. *h*_*t*_ is the summation of $h_{t}^{1}$ and $h_{t}^{2}$.

For the transferred layer, we utilize the knowledge learned from the general domain to initialize the weights of first layer in the multitask bi-directional RNN as following equations.

$$\begin{array}{c} U_{m_{0}}^{1}=U_{g}^{1} \end{array}$$ (7)

$$\begin{array}{c} U_{m_{0}}^{2}=U_{g}^{2} \end{array}$$ (8)

$$\begin{array}{c} W_{m_{0}}^{1}=W_{g}^{1} \end{array}$$ (9)

$$\begin{array}{c} W_{m_{0}}^{2}=W_{g}^{2} \end{array}$$ (10)

where $U_{g}^{1}$, $W_{g}^{1}$, $U_{g}^{2}$, and $W_{g}^{2}$ denote the knowledge learn from the general domain while $U_{m_{0}}^{1}$, $W_{m_{0}}^{1}$, $U_{m_{0}}^{2}$, and $W_{m_{0}}^{2}$ denote the initialization values. In this work, we use a special form of bi-directional RNN, the bi-directional RNN with LSTM cell [30].

**Fig 2:**
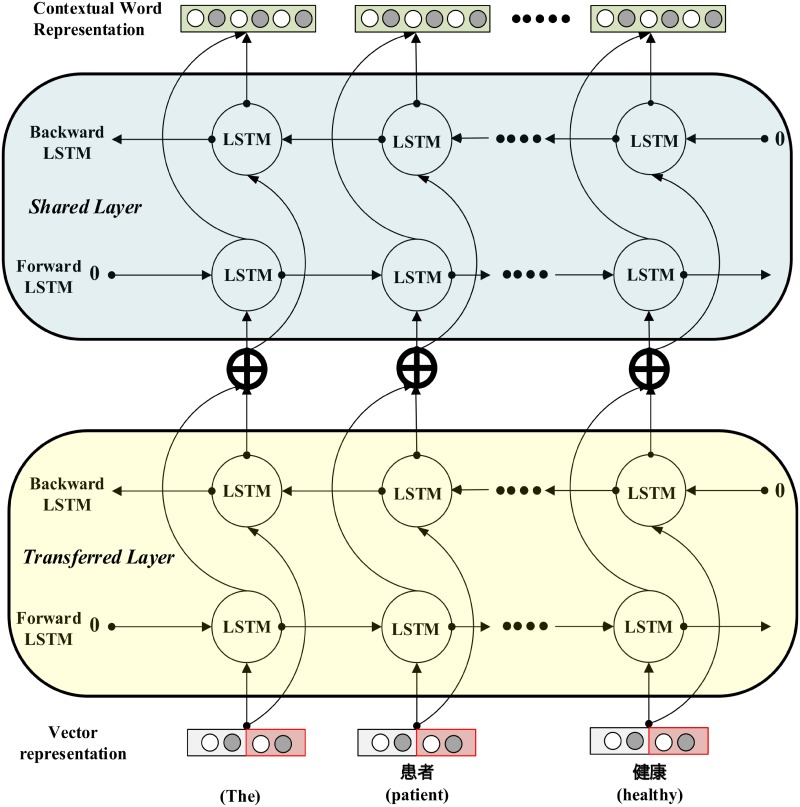
Contextual word representation from vector representation. To extract relevant context information from sentence, bi-directional RNN with LSTM cell is used to extract information from a vector associated with word embedding (red shaded box) and character embedding (white shaded box) to form contextual word representation (green shaded box).

The shared layer contains two consecutive parts. In the first part, each word is represented by a vector developed by Mikolov [31]. The vector is built as a concatenation of word embeddings [32] and character embeddings. Bi-directional RNN with LSTM cell is used to extract features at the character level and represent the features as character embeddings. Word embedding is achieved by *word to vector* [32] representation. Character embeddings and word embeddings are then combined to represent each word in a vector representation. In Fig 2, the vector representation is applied as the input to the transferred layer and shared layer.

**Fig 3:**
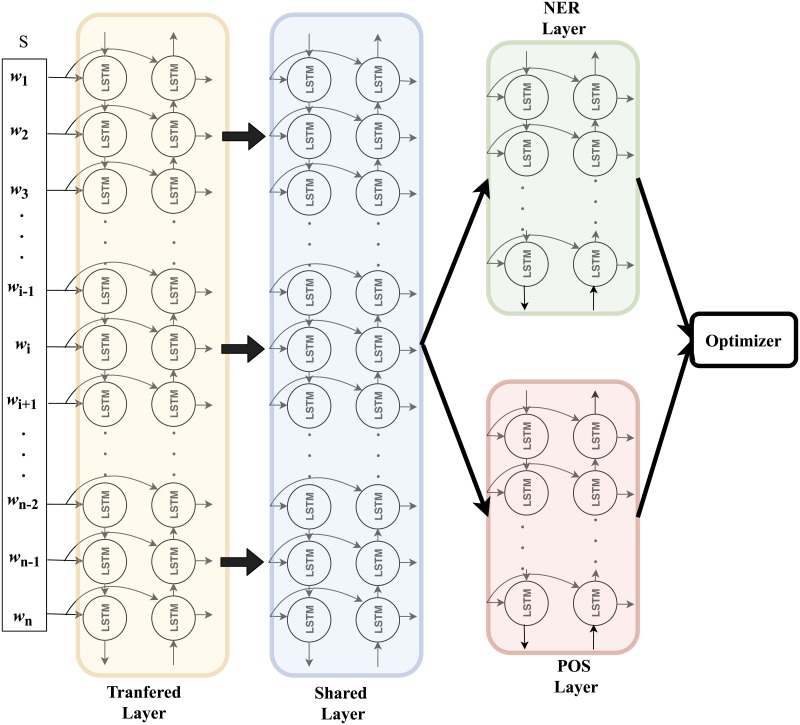
Main architecture of the proposed model that contains transferred layer (yellow shaded box) initialized by deep transfer learning and other three layers, namely, shared layer (blue shaded box), NER layer (red shaded box) and POS layer(green shaded box), where the NER layer and the POS layer are for the task NER and POS, respectively.

Then the outputs (contextual word representations) are shared by two different bi-directional RNN with LSTM cell for two different tasks: parts-of-speech tagging and named entity recognition. These two task layers are trained alternatively so that knowledge from parts-of-tagging task can be used to improve the performance of named entity recognition task. The detailed settings of the proposed model is shown in Table 1 and the corresponding structure is illustrated in Fig 3.

**Table 1:** The proposed network architecture.

Name	Description
Input	Sentences in EMR
Word Embedding	Mikolov model
Character Embedding Layer [21]	150 LSTM cells for each hidden layer, one forward hidden layer andone backward hidden layer, Dropout = 0.5
Transferred layer	300 LSTM cells for each hidden layer, one forward hidden layer and one backward hidden layer, Dropout = 0.5
Shared Layer	300 LSTM cells for each hidden layer, one forward hidden layer and one backward hidden layer, Dropout = 0.5
Parts-of-speech tag (POS) layer	300 LSTM cells for each hidden layer, one forward hidden layer and one backward hidden layer, Dropout = 0.5
Named Entity recognition (NER) Layer	300 LSTM cells for each hidden layer, one forward hidden layer and one backward hidden layer, Dropout = 0.5
Output	Softmax

## Results

### Experimental settings

In this experiment, our proposed model is employed to extract medical information from EMR dataset. The key hyper parameters are: Number of hidden neurons for character embedding layer: 150, Number of hidden neurons for transferred and shared layer: 300, Minibatch size for the case of discharge summary: 50, Minibatch size for the case of progress note: 10, Number of epoch: 100, Optimizer: Adam optimizer, Learning rate: 0.01, Learning rate decay: 0.9. They are determined by trial and error.

### Evaluation metric

Different metrics in terms of micro-average F score (MicroF), macro-average F score (MacroF) [33] and accuracy have been used to evaluate the performance of our proposed model. Macro-average is to calculate the metrics such as Precision, Recall and F-scores independently for each class and then utilize the average of these metrics, whereas Micro-average will aggregate the contributions of all classes to compute the average metrics. Accuracy is calculated by dividing the number of predicted entities that is exactly matched with dataset entities over the total number of entities in the dataset. Generally, we prefer using accuracy to evaluate the model since it shows if the model can recognize the entire entities (each entity may contain multiple words), not just each individual word.

### Experimental results

We evaluate the proposed model with different metrics namely micro average, macro average and accuracy by comparing with classifiers, namely Naive Bayes (NB), Maximum Entropy (ME), Support Vector Machine (SVM), Conditional Random Field (CRF) [24], and deep learning models including Convolutional Neural Network (CNN) [24], single task bi-directional RNN (BRNN), transfer bi-directional RNN (TBRNN) [20], and multitask bidirectional RNN (MBRNN) (Multitask model) [21], where we build multiclass classifiers with these classifiers to resolve NER [24]. BRNN model is selected as the base line model and MBRNN is employed as the state-of-the-art. For TBRNN, we propose a two-step procedure where the first step is to train a shallow bi-directional RNN in the general domain, and the second step is to transfer knowledge from the general domain to train a deeper bi-directional RNN for recognizing medical concepts from Chinese EMRs. For MBRNN, to implement deep multitask learning, a multitask bi-directional RNN model is built for extracting entity terms from Chinese EMR. It can be divided into a shared layer and a task specific layer. Firstly, vector representation of each word is obtained as a concatenation of word embedding and character embedding. Then Bi-directional RNN is used to extract context information from sentence. After that, all these layers are shared by two different task layers, namely the parts-of-speech (POS) tagging task layer and the named entity recognition task layer. These two tasks layers are trained alternatively so that the knowledge learned from named entity recognition task can be enhanced by the knowledge gained from parts-of-speech tagging task.

Firstly, Tables 2 and 3 present comparison performances based on micro average values. The proposed model outperforms compared models, even better than the state-of-the-art. For instance, the MicroF value of our proposed model is improved by 2.55% point and 4.81% point compared to the baseline model (Bi-RNN) and CNN, respectively in terms of results in Table 2. Even compared with the state-of-the-art, we improve the MicroF by 0.14%. Additionally, in Table 3, the MicroF value of our proposed model is improved by 2.23% point and 4.08% point compared to the baseline model (Bi-RNN) and CNN, respectively.

**Table 2:** Comparison results of MicroP, MicroR and MicroF measure on discharge summaries.

Model	MicroP	MicroR	MicroF
Naive Bayes (NB)	78.07	77.91	77.99
Maximum Entropy (ME)	88.81	88.81	88.81
Support Vector Machine (SVM)	90.52	90.52	90.52
Conditional Random Field (CRF) [24]	93.15	93.15	93.15
Convolutional Neural Network (CNN) [24]	88.64	88.64	88.64
Bi-RNN model (BRNN)	90.90	90.90	90.90
Transfer learning Bi-RNN model (TBRNN) [20]	92.25	92.25	92.25
Multitask Bi-RNN model (MBRNN) [21]	93.31	93.31	93.31
Our proposed model	93.45	93.45	93.45

**Table 3:** Comparison results of MicroP, MicroR and MicroF measure on progress notes.

Model	MicroP	MicroR	MicroF
Naive Bayes (NB)	79.42	79.37	79.40
Maximum Entropy (ME)	91.45	91.45	91.45
Support Vector Machine (SVM)	93.07	93.06	93.06
Conditional Random Field (CRF) [24]	94.93	94.02	94.02
Convolutional Neural Network (CNN) [24]	91.13	91.14	91.13
Bi-RNN model (BRNN)	93.58	93.58	93.58
Transfer learning Bi-RNN model (TBRNN) [20]	94.37	94.37	94.37
Multitask Bi-RNN model (MBRNN) [21]	96.65	96.65	96.65
Our proposed model	95.21	95.21	95.21

Since micro average only examine the effectiveness of model from the point of entirety classification, macro average is applied to evaluate the model’s performance from the perspective of different categories of named entities [34]. Table 4 illustrates the comparison performance of NER on discharge summaries. The macro average F-score is improved by 3.20% point compared to the state-of-the-art. The F-measure ranged from 71.43% point to 89.53% point in different categorized entities when it is computed on our proposed model whereas the range is from 57.14% point to 88.61% point when it is computed from the state-of-the-art. The proposed model outperform the state-of-the-art in all comparison of F-measure values. Table 5 shows the comparison results of NER on progress note. The macro average F-score is reduced by 5.12% compared to the state-of-the-art.

**Table 4:** Comparison results of NER on discharge summaries.

Multitask model [21]			
Entity type	Precision	Recall	F-measure
Disease	84.11	84.70	84.40
Symptom	88.08	84.01	86.00
Disease group	43.75	82.35	57.14
Treatment	73.91	82.06	77.77
Test	89.23	87.99	88.61
Macro average	75.82	84.22	78.79
Our proposed model			
Entity type	Precision	Recall	F-measure
Disease	84.31	85.32	84.82
Symptom	87.52	85.14	86.32
Disease group	62.50	83.33	71.43
Treatment	76.20	79.59	77.86
Test	90.16	88.91	89.53
Macro average	80.14	84.46	81.99

**Table 5:** Comparison results of NER on progress notes.

Multitask model [21]			
Entity type	Precision	Recall	F-measure
Disease	94.06	95.07	94.5
Symptom	94.50	90.79	92.61
Disease group	77.27	80.95	79.06
Treatment	88.15	87.19	87.67
Test	92.53	93.36	92.94
Macro average	89.31	89.47	89.37
Our proposed model			
Entity type	Precision	Recall	F-measure
Disease	92.88	91.13	92.00
Symptom	92.79	88.02	90.35
Disease group	59.09	81.25	68.42
Treatment	88.46	90.68	89.56
Test	80.71	81.20	80.95
Macro average	82.78	86.46	84.25

We also check accuracy on discharge summaries and progress notes are given in Tables 6 and 7. It is observed that the overall accuracy is improved by 1.71% point on discharge summary whereas on the progress note it is decreased by 5.78%, compared to the state-of-the-art. It is observed that the best accuracy is enlisted as 90.84% point in test terms and lowest performance is 60.00% point in recognizing disease terms for the case of discharge summary.

**Table 6:** Comparison results (%accuracy) on discharge summaries. TMBRNN is the proposed model.

Model	Entity type					
	Disease	Symptom	Disease group	Treatment	Test	Accuracy
NB	44.82	51.72	N/A	59.00	65.96	58.91
ME	48.32	56.34	34.19	58.80	76.10	65.68
SVM	57.18	62.52	37.22	60.48	80.17	70.46
CRF [24]	77.33	77.83	48.39	77.47	90.05	83.94
CNN [24]	52.80	65.76	40.00	53.14	79.28	68.60
BRNN	73.83	79.35	28.00	67.99	82.63	77.85
TBRNN [20]	74.30	82.60	44.00	68.20	86.79	80.75
MBRNN [21]	76.86	87.22	36.00	71.33	89.20	83.51
TMBRNN	80.37	86.14	60.00	72.17	90.84	85.20

**Table 7:** Comparison results (%accuracy) on progress notes. TMBRNN is the proposed model.

Model	Entity type					
	Disease	Symptom	Disease group	Treatment	Test	Accuracy
NB	69.50	70.09	N/A	41.59	71.85	67.49
ME	71.49	72.37	41.15	52.93	77.58	72.44
SVM	77.77	76.92	21.12	56.36	81.49	76.45
CRF [24]	87.42	87.09	36.06	75.60	90.31	87.22
CNN [24]	76.19	76.65	12.50	51.83	76.65	73.40
BRNN	87.48	87.01	25.00	63.99	83.75	82.72
TBRNN [20]	88.70	88.49	31.25	72.93	86.12	85.43
MBRNN [21]	92.24	94.19	75.00	86.46	92.61	92.13
TMBRNN	89.93	92.02	50.00	77.29	88.94	88.35

**Fig 4:**
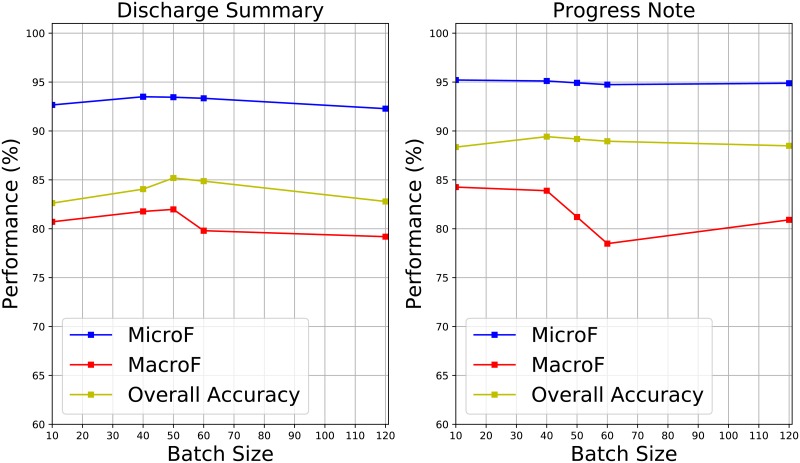
Different overall performance conducted with different batch sizes.

**Fig 5:**
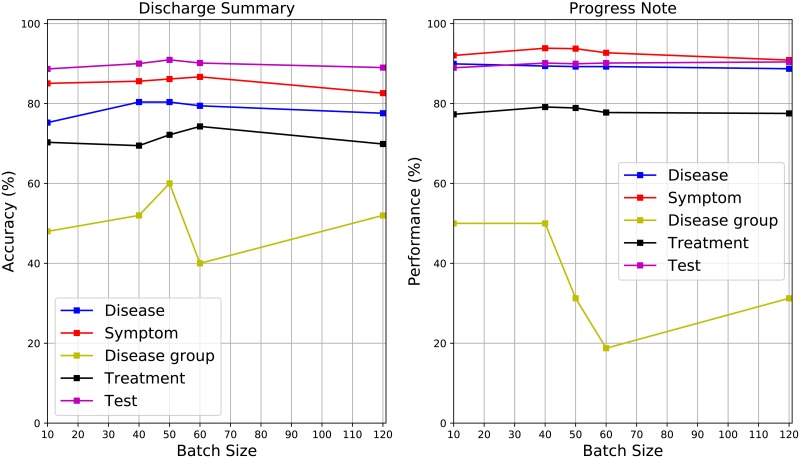
Different accuracies on mining different categories of medical terms with different batch sizes.

Moreover, we also check the affection on performance by different hyper-parameters, namely, batch size and learning rate. Figs 4 and 5 demonstrate different performance generated with different batch sizes, where the learning rate is set as 0.01. In the Fig 4, compared to MicroF and MacroF, the overall accuracies are affected by the selection on batch sizes. In the Fig 5, compared to other entity categories, the accuracies of disease group are changed more significantly. Tables 8 and 9 illustrate different performance generated with different learning rates, where the batch size is set as 50. Compared to the case of batch size, choosing different learning rates affects performance more significantly. Moreover, the smaller the learning rate is, the worse the performance is.

**Table 8:** Comparison results of NER in terms of different learning rates.

Discharge Summary			
Learning Rate	MicroF	MacroF	Overall Accuracy
0.01	93.45	81.98	85.20
0.001	91.22	70.32	78.92
0.0001	83.30	49.65	54.30
Progress Note			
Learning Rate	MicroF	MacroF	Overall Accuracy
0.01	94.92	81.20	89.19
0.001	94.51	76.91	87.60
0.0001	86.79	54.63	64.48

**Table 9:** Comparison results of NER on discharge summaries and progress notes.

Discharge Summary			
Entity type	lr = 0.01	lr = 0.001	lr = 0.0001
Disease	80.37	70.32	41.58
Symptom	86.14	16.00	56.52
Disease group	60.00	70.32	0.00
Treatment	72.17	65.60	40.16
Test	90.94	85.78	62.84
Progress Note			
Entity type	lr = 0.01	lr = 0.001	lr = 0.0001
Disease	89.25	88.84	71.02
Symptom	93.73	91.23	73.80
Disease group	31.25	25.00	0.00
Treatment	78.89	75.23	28.67
Test	89.96	88.88	66.55

## Discussion

In the proposed model, we have been concentrating on improving the accuracy of NER task with limited labeled data. Therefore, we have integrated two kinds of deep learning techniques, namely, deep transfer learning and multitask deep learning. Deep transfer learning is able to utilize transferred knowledge from other task to enhance the prediction accuracy, while multitask deep learning can be viewed as data augmentation that could strengthen the NER performance effectively. However, it introduced some difficulties of building deep learning model. Firstly, it is difficult to determine whether the transferred knowledge would always be effective to enhance the model. For example, in this paper, compared to the multitask deep learning model, the transferred knowledge improves the NER performance in the case of processing discharge summaries whereas reduces the performance for the case of progress notes. In our future research, we will try to leverage the similarity between two domains to judge whether the transferring procedure should be used. Secondly, more training time is required for the proposed model since two task specific layers need to be trained alternatively based on two loss functions. We plan to use a joint loss function and joint optimizer to reduce the training time and improve the accuracy in our future works.

## Conclusion

In this paper, a novel bi-directional RNN model is proposed by combining deep transfer learning and multitask bi-directional LSTM RNN for improving the performance of NER in EMR. The general knowledge extracted from Chinese corpus in the general domain is transferred into the NER task of mining medical terms from Chinese EMR. We initialize the parameters of transferred layer and then build the multitask model with a shared layer and two different task layers, namely parts of speech tagging task layer and named entity recognition task layer. Both transferred layer and shared layer contribute to the improvement of the accuracy of extracting entity information. Evaluation results using real datasets demonstrate the effectiveness of the proposed model.
